# Psychosocial Moderators and Mediators of Sensorimotor Exercise in Low Back Pain: A Randomized Multicenter Controlled Trial

**DOI:** 10.3389/fpsyt.2021.629474

**Published:** 2021-07-29

**Authors:** Pia-Maria Wippert, Daniel Niederer, David Drießlein, Heidrun Beck, Winfried Banzer, Christian Schneider, Marcus Schiltenwolf, Frank Mayer

**Affiliations:** ^1^Sociology of Medicine and Psychobiology, Department of Physical Activity and Health, University of Potsdam, Potsdam, Germany; ^2^Faculty of Health Sciences Brandenburg, University of Potsdam, the Brandenburg Medical School Theodor Fontane and the Brandenburg University of Technology Cottbus, Senftenberg, Germany; ^3^Department of Sports Medicine and Exercise Physiology, Goethe University Frankfurt, Frankfurt am Main, Germany; ^4^Statistical Consulting Unit StaBLab, Ludwig-Maximilians-Universität München, Munich, Germany; ^5^University Hospital Carl Gustav Carus at Technical University Dresden, Dresden, Germany; ^6^Department of Preventive and Sports Medicine, Institute of Occupational, Social and Environmental Medicine, Goethe University Frankfurt, Frankfurt am Main, Germany; ^7^Orthopädiezentrum Theresie, Munich, Germany; ^8^Pain Management, Centre of Orthopaedics and Trauma Surgery, Conservative Orthopaedics and Pain Management, Heidelberg University Hospital, Heidelberg, Germany; ^9^Centre of Sports Medicine, University Outpatient Clinic, University of Potsdam, Potsdam, Germany

**Keywords:** low-back-pain, motor-control-exercise, multidisciplinary-therapy, MiSpEx-network, yellow flags

## Abstract

The effects of exercise interventions on unspecific chronic low back pain (CLBP) have been investigated in many studies, but the results are inconclusive regarding exercise types, efficiency, and sustainability. This may be because the influence of psychosocial factors on exercise induced adaptation regarding CLBP is neglected. Therefore, this study assessed psychosocial characteristics, which moderate and mediate the effects of sensorimotor exercise on LBP. A single-blind 3-arm multicenter randomized controlled trial was conducted for 12-weeks. Three exercise groups, sensorimotor exercise (SMT), sensorimotor and behavioral training (SMT-BT), and regular routines (CG) were randomly assigned to 662 volunteers. Primary outcomes (pain intensity and disability) and psychosocial characteristics were assessed at baseline (M1) and follow-up (3/6/12/24 weeks, M2-M5). Multiple regression models were used to analyze whether psychosocial characteristics are moderators of the relationship between exercise and pain, meaning that psychosocial factors and exercise interact. Causal mediation analysis were conducted to analyze, whether psychosocial characteristics mediate the exercise effect on pain. A total of 453 participants with intermittent pain (mean age = 39.5 ± 12.2 years, f = 62%) completed the training. It was shown, that depressive symptomatology (at M4, M5), vital exhaustion (at M4), and perceived social support (at M5) are significant moderators of the relationship between exercise and the reduction of pain intensity. Further depressive mood (at M4), social-satisfaction (at M4), and anxiety (at M5 SMT) significantly moderate the exercise effect on pain disability. The amount of moderation was of clinical relevance. In contrast, there were no psychosocial variables which mediated exercise effects on pain. In conclusion it was shown, that psychosocial variables can be moderators in the relationship between sensorimotor exercise induced adaptation on CLBP which may explain conflicting results in the past regarding the merit of exercise interventions in CLBP. Results suggest further an early identification of psychosocial risk factors by diagnostic tools, which may essential support the planning of personalized exercise therapy.

**Level of Evidence:** Level I.

**Clinical Trial Registration:** DRKS00004977, LOE: I, MiSpEx: grant-number: 080102A/11-14. https://www.drks.de/drks_web/navigate.do?navigationId=trial.HTML&TRIAL_ID=DRKS00004977.

## Perspective

The effect of sensorimotor exercise on LBP can be moderated by psychosocial factors. Therefore, diagnostic tools that can be applied as a prescreening tool to design a personalized exercise intervention protocol and to optimize the effect of the exercise on LBP are important.

## Introduction

Unspecific chronic low back pain (CLBP, abbreviations in Appendix A) is a worldwide health problem that has not been satisfactorily mitigated with current knowledge and treatment approaches (1). One single solution is not effective; rather, a collective interdisciplinary approach has been recommended by international clinic guidelines (2). The large number of physical and psychological LBP-therapies and their relatively small effects (3–5) have led to the call for individualization of therapies (6) based on diagnostic tools to determine which therapy would be most appropriate for the person (7–9). Unfortunately, solely the personalization of therapies does not solve this problem completely (10). Diagnostic tools may not be precise enough to detect and delineate different areas of risk, (11) or the dose-response relationship between treatment and adaption needs to be investigated more (12).

**Figure 1:**
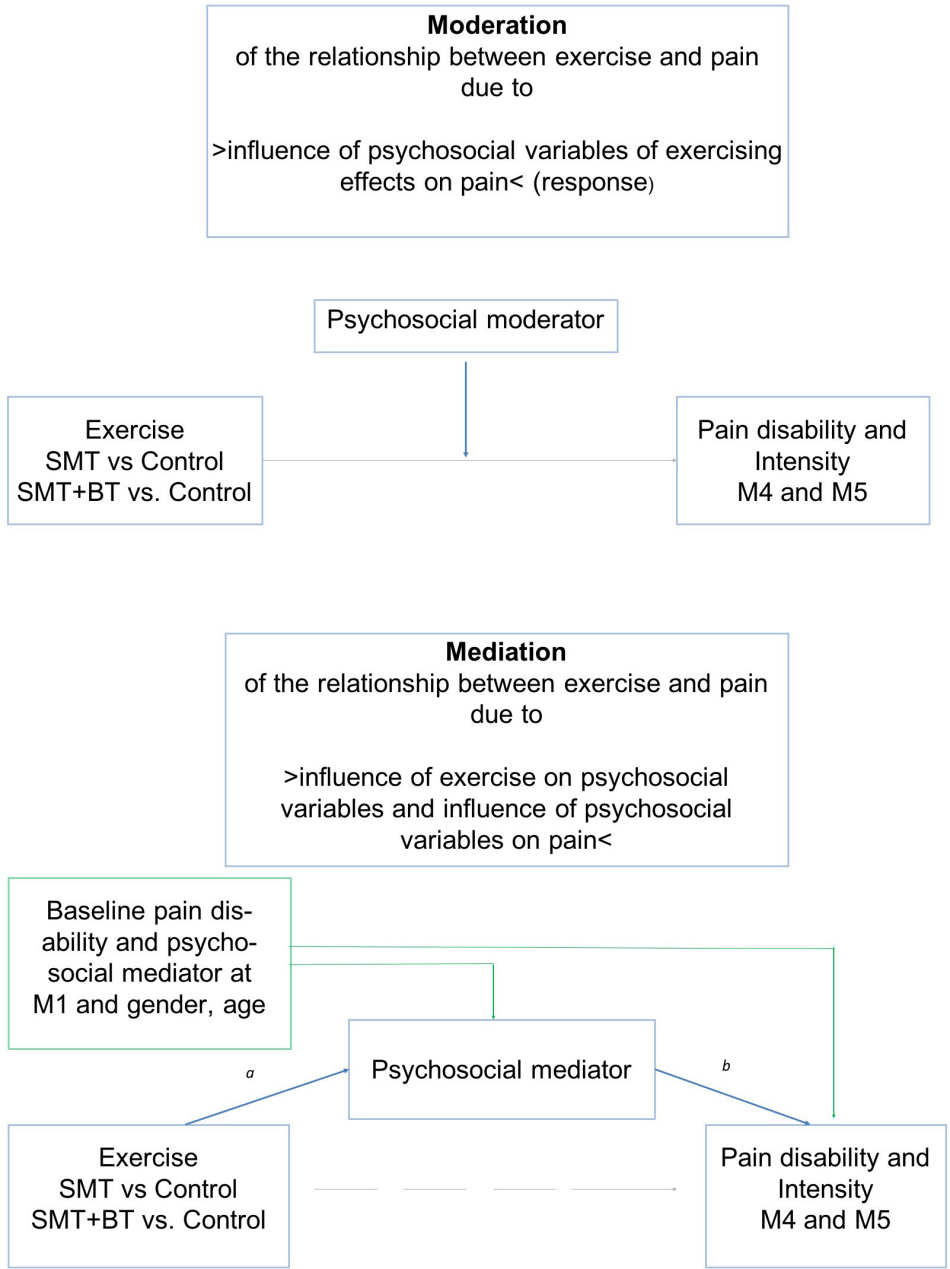
Demonstration of moderation (objective 1) and mediation (objective 2) of psychosocial risk factors on the relationship of exercise and pain. All analyses are controlled for age and sex (mechanisms of interest: blue lines; direct effects: gray lines; possible confounder: green lines).

Exercise can be an important treatment for CLBP (13). However, the effects of exercise on self-reported pain severity and psychological improvements, which only range from small to moderate, are inconsistent (14) and often of weak long-term effects (15). An explanation for this inconsistency may be the impact of psychosocial factors and the reciprocal relationship between exercise and pain. There is evidence that specific psychosocial factors, also called “yellow flags” (16), influence the course of pain. These include distress (17–19), fear avoidance, catastrophizing, and social isolation (20, 21). Some of them have a dosage-related U-shaped relationship with pain (22), a possible explanation for the variation in intervention success of exercise (12, 23). For example, moderate exercise can reduce stress and therefore, it impacts pain (mediation). On the other hand, stress influences the effects of exercise on pain (moderation). Although it is to assume that psychosocial variables account, to some extent, for the relationship between exercise and pain, the direction of these pathways is still unknown (see Figure 1). Theoretically, there are different explanations. For example, stress-related neural pattern alterations influence peripheral nerve function (24), pain transmission (25, 26), and myelination due to metabolic changes (24), all contributing to increased peripheral sensitization (27). Further, psychological stress and depressed mood influence molecular and functional recalibrations among mitochondria and mitochondrial energetics (28), contributing to fatigue and tissue alterations [e.g., nerve growth factor (NGF), innervation density, and bone metabolism (29)] that all tackle the effects of exercising. Therefore, stress-related alterations may influence the sensorimotor system and the adaption to sensorimotor exercise stimuli (30).

This raises the question: to what extent do psychosocial factors alter the response of sensorimotor exercise on pain reduction, the dose-response, and how these psychosocial factors interact with sensorimotor exercise. So far, little is known about the moderation or mediation effect of psychosocial factors on the response to sensorimotor exercise; to the best of our knowledge, there is only one systematic review that stated that psychosocial factors may be moderators of general pain treatments (31), while there are more studies which suggest that fear, catastrophizing, and depression are possible mediators of pain and disability in the context of physical activity treatments (32–34). Further, there is a lack of research that includes various psychosocial factors and investigates the question of moderation and mediation simultaneously. The aim of this randomized controlled trial was to analyze whether there is (1) a moderation of exercise effects on LBP due to psychosocial factors and (2) a mediation of exercise effects on LBP due to psychosocial factors (Figure 1). Study questions were addressed in a three armed intervention design, in which beside a regular therapy group two sensorimotor exercise groups exist. The multimodal module with three additional units [details see (35)] addressed consequently above described psychoneuroendocrinological and molecular pathways: (a) A cognitive distraction module (working memory task) during exercising should target the processing of pain stimuli and their emotional classification within the pain-brain-network; (b) A body scan module should harmonize neuronal activity and neurotransmitter concentration and thus reducing peripheral pain sensitization and transmission; (c) A psychoeducation module inform about pain processing, uncomfortable pain behavior (fear avoidance), and mechanisms of becoming chronic pain.

## Materials and Methods

### Participants

**Figure 2:**
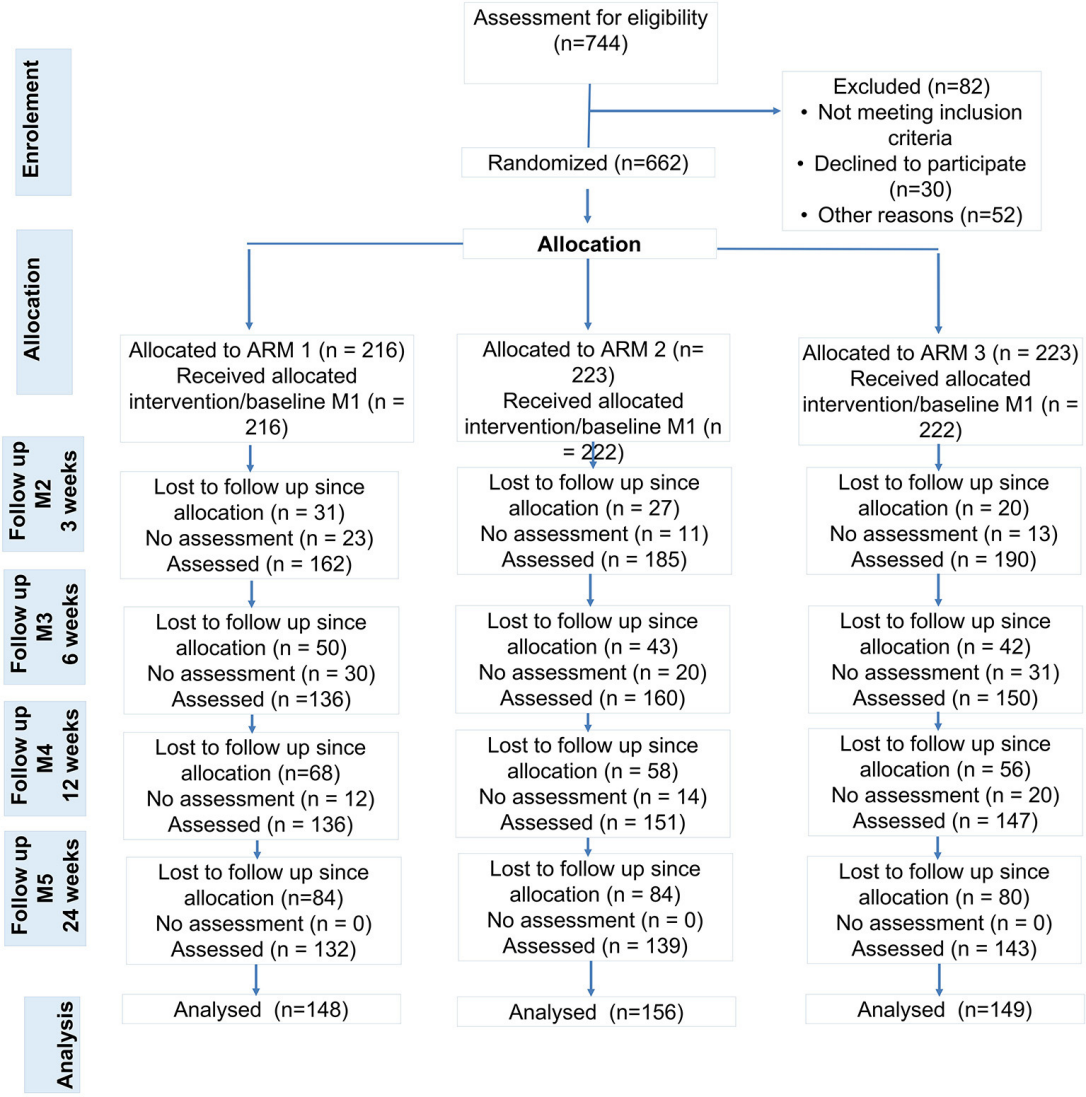
Study Flow (ARM 1: control group, ARM2: unimodal sensorimotor treatment, SMT; ARM 3: multidisciplinary sensorimotor exercise and behavioral treatment, SMT-BT). People with no pain (pain class 0, *n* = 41), athletes (*n* = 11) and people with missing cases at M1, M4 and M5 were excluded from analyses.

Participants were recruited from advertising and while consulting orthopedic outpatient clinics of university sites across Germany for intermittent low back pain for the first time. Study sites are located at different German federal states, offer primary care for general population and for athletes (Center of German Sports Medicine). All five study sites are members of the National Research Network for Medicine in Spine Exercise (MiSpEx, www.mispex.de) and followed the same study routines. Inclusion criteria were as follows: age 18-65 years, non-acute (low) back pain (>20 on a 100-point Visual-Analog Pain Scale). Persons who suffered from acute infections (<7 days), acute back pain (>7 days prior to study), or illnesses/syndromes that contraindicate physical activity (e.g., red flags such as inflammatory, traumatic, or systematic processes), were pregnant, were not able to stand upright independently, and were unable to get up from a lying position or unable to fill out a questionnaire independently were excluded. In total, 744 persons were screened and 662 were included after receiving written and oral information and having signed the informed consent form (enrolment Figure 2). Sample size calculation was based on a feasibility study [α ≤ 0.05; 1-β = 0.999, drop out 30%, power analysis by G^*^Power, (36) effect size f = 0.25, sample size: *n* = 600].

### Design and Procedure

The multicenter study was a single-blind 3-arm randomized controlled trial of a 12-week intervention [Clinical-Trial-Register 05/16/2013, No-DRKS00004977, 06/2013-12/2014, for further information and findings see (37–39)]. Test-retest studies at all study sites guaranteed the validation of measures, methods and intervention routines before the RCT started.

Participants were randomly allocated (n_block_= 18, basis 1:1; www.randomization.com) to a control group (CG, usual care), a unimodal intervention group (SMT, sensorimotor treatment), or a multidisciplinary group (SMT-BT, sensorimotor treatment with behavioral modules) as SMT is one of the most effective exercises for decreasing pain intensity (40–43). An additional intervention description is provided in Appendix B. Exercise diaries secured compliance control.

**Figure 3:**
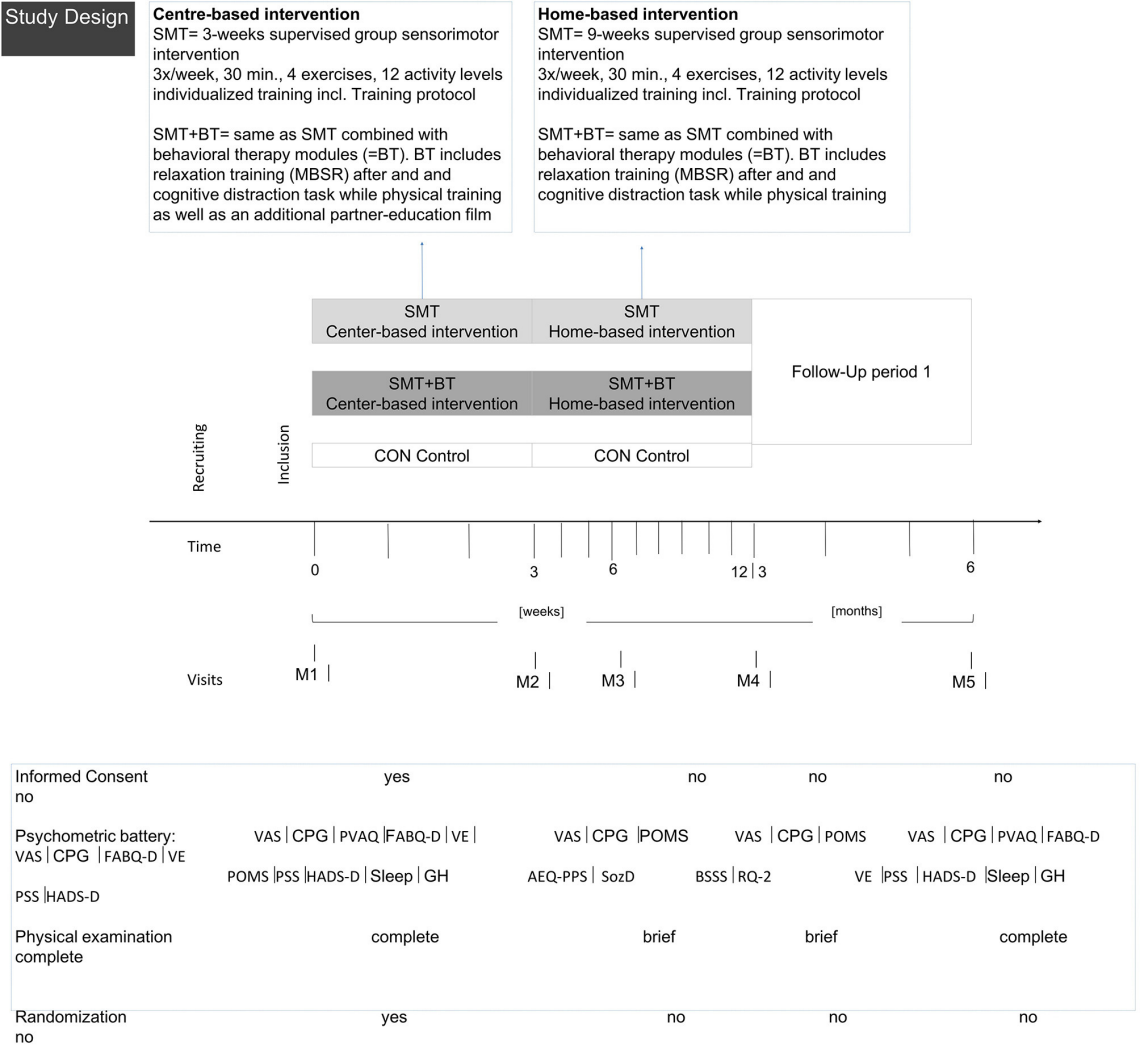
Overview of the study design.

After baseline (M1), measurements were taken after 3 (M2), 6 (M3), 12 (M4), and 24 weeks (M5). Assessments consisted of questionnaires, physical examination, and functional measurements (Figure 3). Study personnel were blinded; participants were instructed not to communicate their group allocation to other participants or the staff. The study was conducted according to the Declaration of Helsinki (ethics approval 01/25/2012, ethics review board University of Potsdam No. 36/2011) and complied with the Consolidated Standards of Reporting Trials (CONSORT).

### Instruments

#### Primary Outcome Criteria

Pain intensity and disability in the past 3 months were assessed by the subscales “characteristic pain intensity” and “subjective pain disability” of the Chronic Pain Grade questionnaire (CPG) (44).

#### Moderators and Mediators

Psychosocial risk factors were captured by different questionnaires and reported separately in three domains (pain experience, stress, and social context).

##### Pain Experience

Anxiety and depression during the previous week were assessed using the Hospital Anxiety and Depression Scale (HADS, two subscales, range: 0 [no anxiety/depression] up to 21 [severe anxiety/depression]; clinical cut off: anxiety >11, depression > 9 points) (45).

Awareness and preoccupation with pain were assessed using the Pain Vigilance and Awareness Questionnaire (PVAQ-D) (46). Higher scores on a single scale (range 0-80) indicate higher levels of awareness and attention to pain.

Fear-avoidance-beliefs were measured using the Fear-Avoidance-Belief-Questionnaire (FABQ-D) (47). Higher scores (range 0-30) indicate stronger fear-avoidance-beliefs (only “Pain caused by physical activity” subscale is presented in this paper).

##### Stress

Subjective stress was measured using the Perceived Stress Scale (PSS) (48). The final score ranges from 0 (no perceived stress) to 40 (high perceived stress), within the last 3 months.

Vital exhaustion (VE), a state of extreme physical fatigue, irritability, and hopelessness “in previous time” was measured with the Maastricht Vital Exhaustion Questionnaire (VE) (49). The final score ranges from 0 to 18 (0-3 = no exhaustion, 4-10 = mild to moderate exhaustion, 11-14 = considerable exhaustion, and <14 points=severe vital exhaustion).

##### Social Environment

Demographics (sex, age, education, employment, and household composition) and lifestyle factors (physical activity, medication, alcohol, and nicotine consumption) were assessed using standardized questions. Social support in the previous week was assessed using the Berliner Social Support Scales (BSSS) (50).

### Statistical Analysis

As study objectives are not an evaluation of treatment main effects (e.g., intention to treat analysis), only complete cases were analyzed. Participants with no pain (defined as Korff pain class 0) and with more than 10 h of physical activity/week (athletes) were excluded from the analysis because of a higher level of structural physiological adaptation at baseline, the different psychosocial life-context, and pain tolerance in comparison to untrained people (51). Missing data imputation were carried out in accordance with questionnaire manuals and American Psychology Association guidelines (52). Missing cases (e.g., missing questionnaire at measurement point) were not respected (complete case analysis).

#### Moderation Analysis

Moderation analysis was performed in R (53), whereby regression models for each psychosocial factor on the dependent outcome, pain intensity and disability, included an interaction between the groups (CG, SMT, SMT-BT) and psychosocial factors (linear interaction models from M1 to M4 and to M5, conservative calculation). Results were presented as the difference between the expected value of the outcome variable of an intervention-group participant and of a control-group participant, given all other parameters held constant. The difference in the effects on pain outcome between the intervention and control groups depended on the value of the moderating factor. Therefore, we present this difference once with the value given by the 20% quantile and a second time by the 80% quantile of each factor (as we are especially interested in people with higher psychosocial risk). For each model, an F-test was performed to test whether the regression coefficients for the interaction significantly improved model fit to the data. All models were controlled for baseline pain, age, sex, physical activity (logarithmic sports variable), and study center.

#### Mediation Analyses

Mediation analysis was conducted with the *mediation* R package (53, 54) under the counterfactual framework (55) which is based on the Imai and colleagues approach to extend Structural Equation Modeling (SEM) by using causal inference methods (56). From the two general analytical approaches for mediation [statistical/traditional (57) and causal], the second one comprehend interactions and assumptions testing's by means of sensitivity analysis (58).

Our causal mediation analysis explored the linear relationship between the intervention (SMT and SMT-BT), a psychosocial factor and the main outcomes (subjective pain disability and pain intensity at M4 and M5). We analyzed the psychosocial factors from the tree yellow flag domains (*pain experience, stress*, and *social environment*) assessed at M4 as mediators.

Estimation of the mediation effect followed two steps. First, two statistical models were fitted separately; (54) to begin with “the mediator model” comprised the mediator (psychosocial factor, e.g., HADS Anxiety) as the dependent variable from the intervention (e.g., SMT). Following, “the outcome model” with pain (subjective pain disability and pain intensity) as a function of the mediator and intervention. In both models, we controlled for baseline measures of pain and mediators (at M1), gender and age. We only used linear regression models, with proper transformations (e.g., logarithmic, square root) when needed to comply with statistical assumptions (e.g., skewness/heteroscedasticity) for these first step.

Second, the mediate function incorporated both models to compute the total (ATE), direct (ADE) and indirect effect (ACME). The ACME is the portion of the total effect of the intervention on the outcome that is conducted though the mediator. While ADE is the remaining effect of the intervention owed to other causal mechanisms (59). In these second step, we applied the non-parametric bootstrap for variance estimation with 1,000 random samples (59).

Further, a test for a potential interaction between intervention and mediator in the “outcome model” was performed. If the interaction test (test.Tmint from mediation package R) showed a positive result (*p*-value <0.05), the treatment–mediator interaction was included.

Only if a mediation effect is detected a sensitivity analysis is planned. The presented approach relies on the “sequential ignorability assumption,” defined as: accounting the observed confounders, the treatment assignment is presumed to be ignorable, namely, independent of potential outcomes and potential mediators (54). When the sequential ignorability assumption is violated a sensitivity analysis is outlined in order to determine the robustness of the ACME estimate (mediation effect) (54, 60).

## Results

### Descriptive

**Figure 4:**
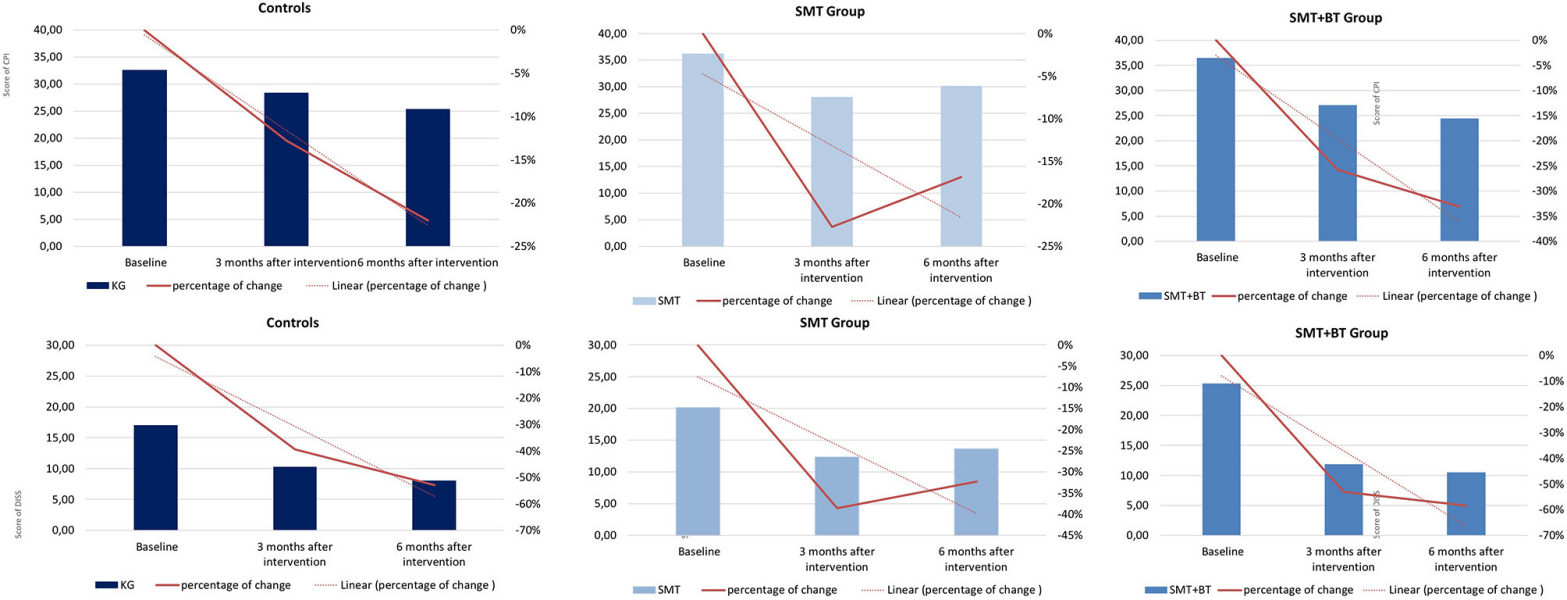
Graphical demonstration of the improvement of pain in percentage from baseline (M1) up to 12 weeks (M4) and 24 weeks (M5) in all groups (pain intensity [above] and pain disability [below]).

Six hundred and sixty volunteers participated, of which 453 (age *M* [*SD*] = 39.5 [12.2] years, *f* = 62%, higher education 45%, pain medication intake 10%, living in partnership 55%) were finally available for the statistical analysis (complete case for M1, M4, and M5, minimum pain CPG-class and exclusion of 11 athletes). The sample examined had moderate pain (for disability: *M* [*SD*] = 20.86 [22.75], and intensity *M* [*SD*] = 35.10 [18.98]), average BMI *M* [*SD*] = 24.30 [3.66], and less comorbidity with a low psychosocial risk at baseline. Only a small portion of the participants showed an elevated psychosocial risk profile, although mostly in the low to moderate range (values for each group see Table 1, Figure 4). Significant differences at baseline was shown for DISS only between SMT+BT and CG (*p* = 0.01) and for BMI between SMT+BT and the other groups. The mean training frequency in the center-based phase was *M* (*SD*) = 2.64 (0.61) times per week and during the home-based phase *M* (*SD*) = 2.40 (0.87) times per week.

**Table 1:** Baseline sample and descriptive characteristics (*M, SD*) of psychosocial factors (separated for each scale/group).

**%, *N* and *p*-value**	***P*** **-value**		**CG**			**SMT**			**SMT+BT**		
Higher Education	0.55		47.00		100	46.61		118	40.52		116
Pain Medication	0.39		7.43		148	12.18		156	10.07		149
Living in Partnership	0.50		52.43		103	54.03		124	59.86		124
Gender	0.64		64.08		103	62.90		124	58.40		125
	***P*** **-value**		**CG**			**SMT**			**SMT+BT**		
**Mean, SD**, ***N**,***and*****p*****-value**			***M***	***SD***	***N***	***M***	***SD***	***N***	***M***	***SD***	***N***
Age	0.16		37.94	12.81	103	39.33	11.34	123	41.02	12.46	125
BMI	0.01		23.79	3.41	127	23.97	3.35	129	25.14	4.04	128
DISS	0.01		17.05	21.93	146	20.16	20.96	153	25.35	24.64	147
CPI	0.15		32.59	19.47	147	36.17	18.68	155	36.49	18.68	147
	**Total Group**		**CG**			**SMT**			**SMT+BT**		
**Mean, SD, and quantiles**	**Low 20%**	**High 80%**	***M***	***SD***	***N***	***M***	***SD***	***N***	***M***	***SD***	***N***
Fear Avoidance Activity FABQ^a^	7.00	18.00	12.56	6.48	142	12.46	5.82	147	12.85	5.27	140
HADS anxiety^b^	3.00	8.00	5.17	3.04	144	5.60	3.45	153	5.28	3.05	144
HADS depression	1.00	6.00	3.69	2.99	144	4.03	3.03	151	3.81	2.95	145
Perceived stress scale PSS^c^	11.00	21.00	16.09	6.47	139	16.51	6.05	146	15.96	5.77	139
Vital exhaustion VE^d^	2.00	12.00	7.00	4.87	144	8.07	4.85	150	7.53	5.13	143
Pain Vigilance Avoidance PVAQ^e^	28.00	49.00	37.27	13.02	146	39.03	12.13	154	38.13	12.47	147
Mood displeasure POMS^f^	2.00	13.00	7.11	6.77	146	8.70	7.41	152	7.66	7.17	146
Mood depression POMS	1.00	17.00	9.41	10.78	142	10.69	11.95	152	10.06	12.74	145
Perceived Social Support BSSS^g^	3.25	4.00	3.63	0.53	87	3.64	0.43	106	3.62	0.51	94
Received Social Support BSSS	2.82	3.80	3.27	0.55	87	3.25	0.48	106	3.36	0.52	94
Satisfaction with Social Support BSSS	3.00	4.00	3.72	0.50	86	3.73	0.53	106	3.71	0.58	94

*Additional presentation of 20% and 80% quantiles of psychosocial risk in total group*.

a *FABQ, pain related cognitions scale: fab _activity (caused by bodily activity, score 0–30) at M1*;

b *HADS-D, Anxiety and Depression (score 0–21) at M1*;

c *PSS, Perceived Stress Scale (score 1–40) at M1*;

d *Vital Exhaustion questionnaire (score 0–18) at M1*,

e *Pain Vigilance and Avoidance Questionnaire (score 0–80) at M1*;

f *POMS displeasure (score 0–84), POMS depression (score 0–42) last 24 h at M1*;

g *BSSS (perceived, received support score 1–4) at M3*.

### Role of Psychosocial Moderators on the Intervention Effect on Pain

#### Pain Experience

There was no significant moderation of the intervention effects by fear avoidance regarding physical activity (kinesiophobia), by pain vigilance-avoidance and by anxiety after the intervention (M4). In contrast, depressive mood (POMS) and depressive symptomatology (HADS) at baseline were systematic moderators of the intervention effects on CPI. For the multidisciplinary group (SMT-BT), for example, the expected CPI value in M4 was 8.62 points lower than the expected CPI in the control group for patients with a HADS depression value of 6.00. Furthermore, depressive symptoms (HADS) significantly moderated pain disability (Table 2). By subtracting from the value of SMT-BT the value of SMT for a given quantile one is able to derive the expected difference in CPI/DISS between SMT-BT and SMT patients. For example the expected CPI value in M4 for a patient in SMT-BT with a HADS depression value of 6.00 (80% quantile) is −8.62 - (-4.21) = −4.41 points i.e. 4.41 points lower than for a patient in SMT. Remark: A negative difference implies a lower, a positive difference a higher value for SMT-BT compared to SMT.

**Table 2:** Interactions-metrics of intervention effects for subjective pain disability and pain intensity 12 weeks (M4) after the intervention (20%, 80% quantiles).

**M1 to M4**	**Pain disability^a^**					**Pain intensity^a^**				
**Psychosocial factor**	**Low: 20%**		**High: 80%**			**Low: 20%**		**High: 80%**		
	**SMT**	**SMT_BT**	**SMT**	**SMT_BT**	***F*-value (*p*)**	**SMT**	**SMT_BT**	**SMT**	**SMT_BT**	***F*-value (*p*)**
BSSS_Sup_perceived	0.46^b^	−2.88	7.54	6.31	1.42 (0.24)	−0.23	−0.87	3.74	4.35	0.67 (0.51)
BSSS_Sup_received	−0.94	−0.70	10.44	5.10	1.46 (0.24)	−1.60	0.31	6.92	5.69	1.24 (0.29)
BSSS_Sup_satisfaction	−5.08	−9.79	6.97	5.31	3.16 (0.05^*^)	−1.91	−3.68	3.94	3.87	1.15 (0.32)
FABQ_activity^b^	6.98	0.73	−1.48	−3.35	1.18 (0.31)	−1.60	−3.95	−0.66	−1.31	0.12 (0.89)
HADS anxiety^c^	3.67	0.05	2.93	0.61	0.04 (0.96)	1.16	0.64	−3.51	−4.17	0.86 (0.43)
HADS depression	4.65	1.58	1.94	−1.38	0.23 (0.80)	1.22	4.57	−4.21	−8.62	3.77 (0.03^*^)
POMS displeasure	6.24	3.93	0.06	−5.35	1.82 (0.17)	0.82	2.02	−3.98	−7.68	2.05 (0.13^#^)
POMS depression	10.55	5.08	−1.73	−3.21	5.04 (0.01)^*^	5.00	3.38	−4.86	−4.26	4.11 (0.02)^*^
PSS^d^	6.96	1.43	−0.98	0.63	1.46 (0.24)	0.92	1.83	−4.80	−5.07	1.26 (0.29)
VE^e^	5.71	0.77	1.05	0.32	0.34 (0.71)	4.27	6.89	−6.66	−8.87	3.97 (0.02)^*^
PVAQ^f^	5.91	0.57	0.81	−1.95	0.57 (0.57)	−2.16	−0.58	−0.39	−4.16	0.69 (0.51)

# *p < 0.1*,

* *p < 0.05*;

*Multiple regression models for the interaction between intervention group and psychosocial moderators (separate models) for the outcome pain intensity (Pain Disability DISS and Pain intensity CPI) to M4. Each model was adjusted for baseline pain value, gender, age study center and logarithmised sport intensity to M1*.

a *CPG pain scales: CPI characteristic pain intensity and DISS: subjective disability (score 0–100)*,

b *FABQ: pain related cognitions scale: fab _activity (caused by bodily activity, score 0-30)*,

c *HADS-D: Anxiety and Depression (score 0–21)*,

d *PSS: Perceived Stress Scale (score 1–40)*,

e *Vital Exhaustion questionnaire (score 0–18)*,

f *Pain Vigilance and Avoidance Questionnaire (score 0–80), POMS displeasure (score 0–84), POMS depression (score 0–42) last 24 h, BSSS (perceived, received support score 1–4)*.

b *Example: The value 0.46 describes the difference between SMT and CT for the expected value of pain disability calculated for patients with a low value of BSSS_Sup_perceived of 3.25 (see Table 1) which is the 20 % quantile of this psychosocial factor. In other words: For a patient in SMT we expect a 0.46 higher value of Pain Disability than for a patient in CG both having a low BSSS_Sup_perceived value of 3.25 and all other parameters identical*.

*Differences between SMT-BT and SMT can be calculated via subtraction of the two values themselves as exemplarily described in the text*.

Regarding the sustainability of the programs (M5), it was shown that depressive symptomatology is a significant moderator of the intervention effects (8.81 CPG points in pain intensity in the multidisciplinary group); no improvement was observed in the SMT group. The opposite was true for the significant moderation due to anxiety: participants who were anxious at the beginning of the intervention and improved in the meantime, deteriorated again, especially in the SMT-BT group (Table 3).

**Table 3:** Interactions-metrics of intervention effects for subjective pain disability and pain intensity 24 weeks (M5) after the intervention (20%, 80% quantiles).

**M1 to M5**	**Pain disability^a^**					**Pain intensity^a^**				
**Psychosocial factor**	**Low: 20%**		**High: 80%**			**Low: 20%**		**High: 80%**		
	**SMT**	**SMT_BT**	**SMT**	**SMT_BT**	***F*-value p**	**SMT**	**SMT_BT**	**SMT**	**SMT_BT**	***F*-value p**
BSSS_Sup_perceived	5.26	0.63	2.00	0.39	0.20 (0.82)	8.61	−2.45	−4.75	−3.09	3.84 (0.02)^*^
BSSS_Sup_received	4.43	2.55	2.28	−0.70	0.14 (0.88)	4.61	0.97	−3.20	−5.23	0.93 (0.40)
BSSS_Sup_satisfaction	6.76	7.00	4.66	1.45	0.30 (0.74)	8.75	3.47	−0.30	−3.33	1.12 (0.33)
FABQ_activity^b^	6.29	0.36	0.96	−2.77	0.59 (0.56)	0.67	−4.64	8.73	2.52	1.37 (0.26)
HADS anxiety^c^	8.25	0.20	0.05	2.93	3.44 (0.03)^*^	7.94	1.93	0.04	−3.30	1.65 (0.20)
HADS depression	5.15	2.78	2.26	−3.74	0.73 (0.48)	3.24	4.20	3.45	−8.81	3.27 (0.04)^*^
POMS displeasure	4.16	1.09	3.28	−1.69	0.15 (0.86)	3.37	2.29	2.50	−6.26	1.45 (0.24)
POMS depression	9.09	1.51	0.12	0.14	2.30 (0.10)^#^	7.50	2.67	−0.10	−4.68	1.43 (0.24)
PSS^d^	4.99	−1.47	2.84	2.58	0.72 (0.49)	1.15	−0.43	4.69	−2.32	0.57 (0.57)
VE^e^	4.15	0.61	3.21	−0.59	0.02 (1.00)	0.93	3.87	4.74	−6.23	2.26 (0.11)^#^
PVAQ^f^	4.39	1.18	2.78	−2.47	0.29 (0.75)	2.89	1.18	4.16	−4.84	1.14 (0.32)

# *p < 0.1*,

* *p < 0.05*;

*Multiple regression models for the interaction between intervention group and psychosocial moderators (separate models) for the outcome pain intensity (Pain Disability DISS and Pain intensity CPI) to M5. Each model was adjusted for baseline pain value, gender, age study center, and logarithmised sport intensity to M1*.

a *CPG pain scales: CPI characteristic pain intensity and DISS: subjective disability (score 0–100)*,

b *FABQ: pain related cognitions scale: fab _activity (caused by bodily activity, score 0–30)*,

c *HADS-D: Anxiety and Depression (score 0–21)*,

d *PSS: Perceived Stress Scale (score 1–40)*,

e *Vital Exhaustion questionnaire (score 0–18)*,

f *Pain Vigilance and Avoidance Questionnaire (score 0–80), POMS displeasure (score 0–84), POMS depression (score 0–42) last 24 h, BSSS (perceived, received support score 1–4)*.

#### Stress

**Figure 5:**
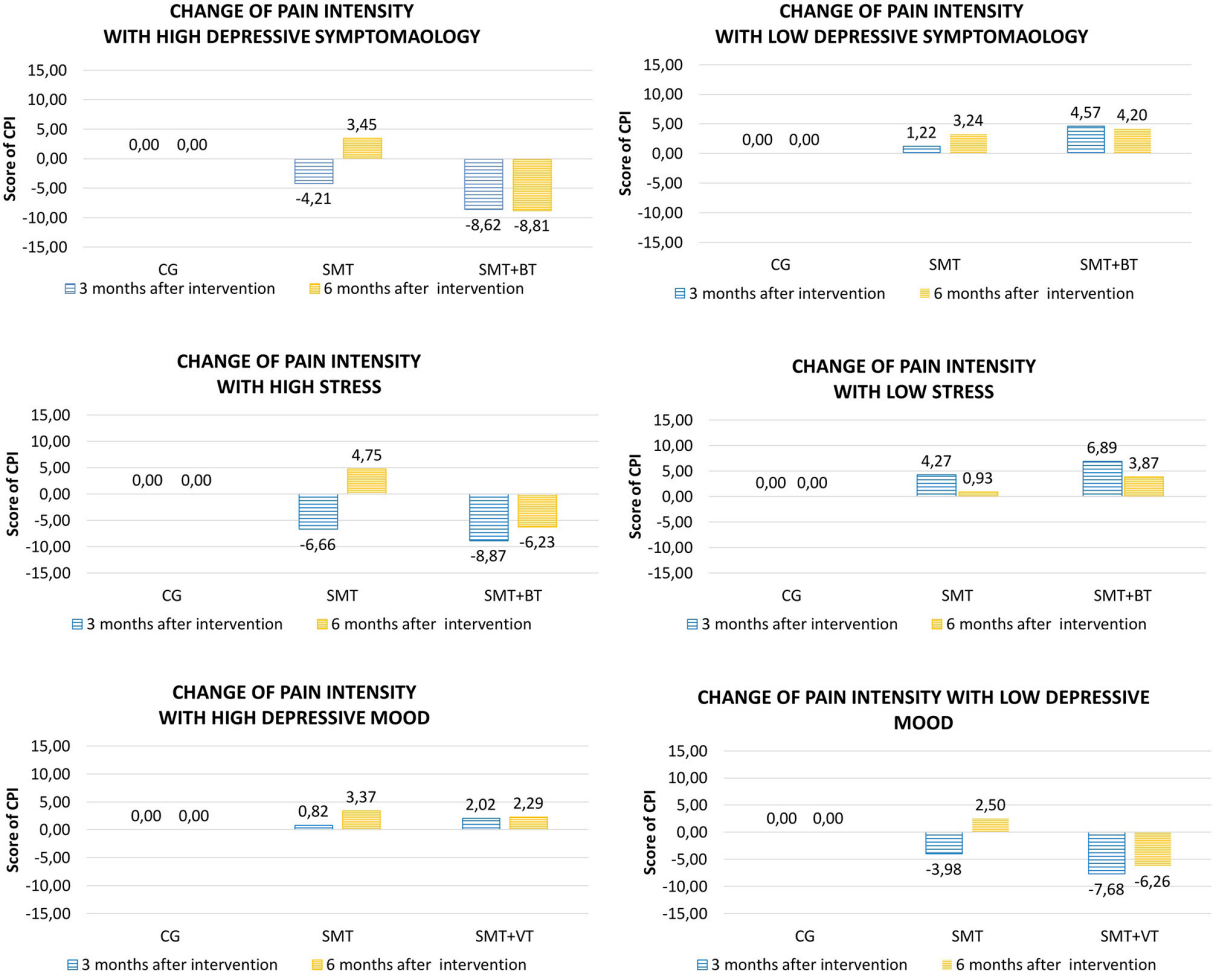
Graphical presentation of the significant psychosocial moderators of Tables 2, 3.

A significant moderation of the intervention effects on CPI after the intervention (M4) was observed by vital exhaustion. This means that highly exhausted participants, each with a VE value of 12 at baseline, would improve their CPI intervention effect scores in comparison to a control group participant around 8.87 CGP points in the SMT-BT group and around 6.66 points in the SMT group. No significant moderation was observed for the Perceived Stress Scale (Table 2 and Figure 5). At follow-up, there was no sustainable effect due to domain stress, although vital exhaustion showed a trend toward improvement of pain intensity in the multidisciplinary group (Table 3).

#### Social Environment

Satisfaction with social support was a moderator of the intervention effects on pain disability. Social support in the center-based treated control group significantly moderated the intervention effects on pain disability in comparison to the home-based intervention groups (Table 2). Regarding the sustainability of the intervention effects, the opposite was observed: perceived social support was a significant moderator of the improvement of intervention effects on pain intensity for the home-based intervention groups (Table 3).

### Role of Psychosocial Mediators on the Intervention Effect on Pain

None of the models showed a significant average total effect (overall intervention effect) and no psychosocial factor played a significant role as a mediator for the relationship between intervention (SMT or SMT-BT) and subjective pain disability (at M4 or M5, Table 4) as well as CPI (at M4 or M5, Table 5).

**Table 4:** Effect decomposition for each single-mediator model.

	**Linear regression models estimates**		**Estimation of the mediation effect**			**Linear regression models estimates**		**Estimation of the mediation effect**		
**Analysis**	**Intervention-mediator effect (path a)**	**Mediator-outcome effect (path b)**	**ATE**	**ADE**	**ACME**	**Intervention-mediator effect (path a)**	**Mediator-outcome effect (path b)**	**ATE**	**ADE**	**ACME**
**M4**	**SMT vs. Control**					**SMT-BT vs. Control**				
Fear Avoidance Activity FABQ	1.97* (0.38 to 3.55)	0.03 (−0.01 to 0.07)^a^	0.07 (−0.31 to 0.43)	0.01 (−0.40 to 0.36)	0.06 (−0.03 to 0.21)	2.36* (0.56 to 4.17)	0.06* (0.02 to 0.09)^a^	−0.09 (−0.53 to 0.34)	−0.23 (−0.65 to 0.21)	0.14 (−0.02 to 0.32)
HADS Anxiety	−0.22* (−0.38 to −0.06)	0.18 (−0.25 to 0.62)^a^	−0.01 (−0.42 to 0.66)	0.03 (−0.40 to 0.37)	−0.04 (−0.18 to 0.07)	–	–	–	–	–
HADS depression	–	–	–	–	–	–	–	–	–	–
Perceived Stress	−1.98* (−3.56 to −0.39)	0.02 (−0.02 to 0.06)^a^	−0.06 (−0.44 to 0.33)	−0.02 (−0.42 to 0.39)	−0.04 (−0.17 to 0.05)	−1.22 (−2.87 to 0.42)	−0.00 (−0.04 to 0.04)^a^	0.06 (−0.36 to 0.49)	0.06 (−0.36 to 0.49)	−0.00 (−0.07 to 0.08)
Vital Exhaustion	−1.31* (−2.49 to −0.12)	0.02 (−0.03 to 0.07)^a^	0.09 (−0.29 to 0.47)	0.12 (−0.29 to 0.53)	−0.03 (−0.12 to 0.04)	−0.12 (−0.39 to 0.15)^b^	0.37* (0.10 to 0.63)^a^	0.05 (−0.37 to 0.49)	0.10 (−0.31 to 0.55)	−0.05 (−0.19 to 0.06)
Pain Vigilance Avoidance PVAQ	−0.37 (−3.18 to 2.43)	0.02 (−0.001 to 0.04)^a^	0.01 (−0.38 to 0.37)	0.02 (−0.36 to 0.37)	−0.01 (−0.08 to 0.05)	0.51 (−2.42 to 3.45)	0.03* (0.008 to 0.06)^a^	−0.03 (−0.43 to 0.39)	−0.04 (−0.45 to 0.38)	0.02 (−0.08 to 0.13)
	**Linear regression models estimates**		**Estimation of the mediation effect**			**Linear regression models estimates**		**Estimation of the mediation effect**		
**Analysis**	**Intervention-mediator effect (path a)**	**Mediator-outcome effect (path b)**	**ATE**	**ADE**	**ACME**	**Intervention-mediator effect (path a)**	**Mediator-outcome effect (path b)**	**ATE**	**ADE**	**ACME**
**M5**	**SMT vs. Control**				**SMT-BT vs. Control**				
Fear Avoidance Activity FABQ	2.10* (0.48 to 3.71)	−0.00 (−0.05 to 0.05)^a^	0.19 (−0.23 to 0.59)	0.20 (−0.22 to 0.62)	−0.01 (−0.14 to 0.09)	–	–	–	–	–
HADS Anxiety	−0.18* (−0.34 to −0.01)^a^	0.50* (0.04 to 0.96)^a^	0.19 (−0.22 to 0.60)	0.29 (−0.10 to 0.68)	−0.10 (−0.25 to 0.00)	–	–	–	–	–
HADS depression	–	–	–	–	–	–	–	–	–	–
Perceived Stress	–	–	–	–	–	−0.78 (−2.51 to 0.94)	0.04 (−0.05 to 0.12)^b^	−0.38 (−1.13 to 0.38)	−0.36 (−1.11 to 0.40)	−0.03 (−0.16 to 0.08)
Vital Exhaustion	−1.28 (−2.60 to −0.04)	0.02 (−0.04 to 0.08)^a^	0.19 (−0.18 to 0.61)	0.22 (−0.15 to 0.64)	−0.03 (−0.11 to 0.04)	–	–	–	–	–
Pain Vigilance Avoidance PVAQ	0.72 (−2.12 to 3.56)	0.00 (−0.02 to 0.03)^a^	0.15 (−0.24 to 0.56)	0.15 (−0.26 to 0.56)	0.00 (−0.04 to 0.05)	0.89 (−2.32 to 4.09)	0.02 (−0.01 to 0.04)^a^	−0.24 (−0.70 to 0.21)	−0.26 (−0.72 to 0.18)	0.02 (−0.04 to 0.14)

Indirect, direct, and total effects of the mediation models on the outcome subjective pain disability at M4 and M5. p < 0.05^*^;

a *Logarithmic transformation*,

b *Square-root transformation, - Violation of linear regression assumptions (Skewness, kurtosis, Link function or Heteroscedasticity) even after transformations. The results of the linear regression of these models would not be interpretable, so not displayed*.

**Table 5:** Effect decomposition for each single-mediator model.

	**Linear regression models estimates**		**Estimation of the mediation effect**			**Linear regression models estimates**		**Estimation of the mediation effect**		
**Analysis**	**Intervention-mediator effect (path a)**	**Mediator-outcome effect (path b)**	**ATE**	**ADE**	**ACME**	**Intervention-mediator effect (path a)**	**Mediator-outcome effect (path b)**	**ATE**	**ADE**	**ACME**
**M4**	**SMT vs. Control**					**SMT-BT vs. Control**				
Fear Avoidance Activity FABQ	–	–	–	–	–	–	–	–	–	–
HADS Anxiety	–	–	–	–	–	–	–	–	–	–
HADS depression	–	–	–	–	–	–	–	–	–	–
Perceived Stress	–	–	–	–	–	–	–	–	–	–
Vital Exhaustion	–	–	–	–	–	–	–	–	–	–
Pain Vigilance Avoidance PVAQ	–	–	–	–	–	0.53 (−2.38 to 3.43)	0.43* (0.13 to 0.72)	−1.12 (−6.39 to 3.77)	−1.35 (−6.40 to 3.69)	0.23 (−1.12 to 1.92)
	**Linear regression models estimates**		**Estimation of the mediation effect**			**Linear regression models estimates**		**Estimation of the mediation effect**		
**Analysis**	**Intervention-mediator effect (path a)**	**Mediator-outcome effect (path b)**	**ATE**	**ADE**	**ACME**	**Intervention-mediator effect (path a)**	**Mediator-outcome effect (path b)**	**ATE**	**ADE**	**ACME**
**M5**	**SMT vs. Control**					**SMT-BT vs. Control**				
Fear Avoidance Activity FABQ	2.34* (0.69 to 3.99)	0.41 (−0.17 to 0.98)	2.26 (−2.93 to 7.26)	1.40 (−4.08 to 6.71)	0.86 (−0.56 to 2.57)	3.24* (1.21 to 5.27)	0.51* (0.01 to 1.02)	−1.85 (−7.72 to 4.21)	−3.85 (−9.59 to 2.32)	2.00 (−0.11 to 4.60)
HADS Anxiety	–	–	–	–	–	–	–	–	–	–
HADS depression	–	–	–	–	–	–	–	–	–	–
Perceived Stress	−1.87* (−3.55 to −0.19)	0.44 (−0.17 to 1.04)	1.84 (−3.73 to 7.43)	2.77 (−3.31 to 8.25)	−0.93 (−2.45 to 0.50)	−0.80 (−2.53 to 0.93)	0.46 (−0.21 to 1.13)	−1.80 (−7.73 to 4.21)	−1.44 (−7.44 to 4.47)	−0.36 (−1.54 to 0.59)
Vital Exhaustion	–	–	–	–	–	−0.21 (−0.50 to 0.08)^b^	0.43 (−0.02 to 0.87)^b^	−0.35 (−1.06 to 0.32)	−0.27 (−0.94 to 0.37)	−0.08 (−0.28 to 0.05)
Pain Vigilance Avoidance PVAQ	0.76 (−2.07 to 3.59)	0.05* (0.01 to 0.08)^b^	0.20 (−0.38 to 0.72)	0.17 (−0.40 to 0.67)	0.03 (−0.08 to 0.20)	0.81 (−2.40 to 4.01)	0.42* (0.09 to 0.74)	−1.93 (−7.47 to 3.47)	−2.27 (−7.53 to 2.75)	0.34 (−0.81 to 2.16)

Indirect, direct, and total effects of the mediation models on the outcome pain intensity at M4 and M5. p < 0.05^*^;

a *Logarithmic transformation*,

b *Square-root transformation, - Violation of linear regression assumptions (Skewness, kurtosis, Link function or Heteroscedasticity) even after transformations. The results of the linear regression of these models would not be interpretable, so not displayed*.

## Discussion

This paper addressed the question whether psychosocial risk factors are moderators or mediators of the effects of sensorimotor exercise intervention on LBP.

### Moderation

#### Role of Psychosocial Moderators

Results show a moderation of the intervention effects on pain syndromes in both exercise interventions compared to the controls in the short-term and further for the multidisciplinary group in the long-term. The magnitude of depressive symptomatology, vital exhaustion, and social support is a moderator of the relationship between exercise efficacy and pain. Results suggest that people with high depressive symptomatology should be better treated with multidisciplinary interventions, for which moderation amplitude reaches a clinically relevant reduction in pain intensity and disability (61–63). Further of clinical relevance was the moderation of sustainability effects of the multidisciplinary therapy due to a depressive symptomatology. While the moderation of exercise effects through vital exhaustion for the short- and long-term efficacy were comparable, the sustainability for anxiety decreased, but without clinical relevance. In summary, both of the identified moderators “depression” and “vital exhaustion” may limit long-term adaptions of the sensorimotor system to exercise stimuli regarding pain.

The reported pain syndromes of the participants may be best sub-summarized in the ICD-11 category nociplastic/algopathic/nocipathic pain, (64–66) in which pain syndromes can be explained by a central sensitization including altered nociception. Further, the two identified moderators that may influence exercise efficacy in terms of pain reduction can be sub-summarized to a “symptom triad” often described in patients with non-specific pain, which is, a co-occurrence of depression, fatigue, and pain. Different research groups try to explain this phenomenon and essentially points to an overload or “catabolic state” of the body (18, 67, 68).

However, several explanations are given for this. As introduced, a stress-induced catabolic state may alter myelination (24) innervation density (29), peripheral nerve function (24), pain transmission (25, 26), contributing to increased peripheral sensitization (27) and possibly altering sensorimotor system response. Also, the changes in neurotransmitter concentration and inflammatory response (e.g., neuropeptides, norepinephrine) (69) may be a profound explanation for the presented results (19, 70), in which vital exhaustion and depressive symptoms mainly moderated exercised induced pain intensity outcomes. Exercise stimuli may be differently processed depending on the exercise dose/intensity.

#### Moderation Within Specific Treatments

Furthermore, it is known that people with an altered nociceptive function respond better to therapies targeting central rather than peripheral pain mechanisms (64); the presented results confirm this by showing a higher benefit from the multidisciplinary intervention for people suffering on depressive symptoms. As the multimodal intervention triggers an activation of executive functions while exercising, the central processing of pain and emotional classification will be altered (71). Effects of an unimodal sensorimotor exercising such a regulation of neural circuits influencing mitochondrial energetics, can be moderated by fatigue as it is seen in the results as well. This gives notice about the catabolic state of the body limiting the adaptive capacity to neuromuscular exercise stimuli. In both cases, the implication for clinicians might be, to tailor exercise therapy to individual patient needs as far as possible.

#### Moderators Within Clinical Setting

As observed by many therapists, the ambulatory setting of regular routines leads to a specific comfortable effect, measured by a significant moderation of the treatment effects by social satisfaction in the control group during the intervention. Interestingly, after the end of the intervention, perceived support in exercising groups moderated the sustainability within their home-based social environment. This decisive factor, to give the patient the feeling of being “perceived” and “accepted,” may be implemented quite inexpensively in therapeutic and preventive everyday life.

### Mediation

No mediation of fear avoidance, anxiety, stress, vital exhaustion, or pain vigilance on exercise effects was observed. Pain-killing medication and lower body mass index, potential positive treatment effect modifiers in other studies (72), had no influence (pain-killing medication). Although results are in line with a recently published trial (73), some studies suggesting that effects on pain can be mediated by depression, fear avoidance or pain catastrophizing (32, 33, 74) and further that these mediation effects can also play a role within a physical activity context (33, 34). A closer look to these studies give notice, that mostly pain disability is chosen as potential outcome and that exercise therapy is not specified. Studies examine mediation processes of physical activity in general, which can be influenced by age, gender, weight and social setting and are rarely controlled in the analysis. Further the methodological quality of most of these studies were low (32). Only one study showing a pain catastrophizing mediation effect after exercising referring to concrete exercise routines (aerobic and endurance training) (34). Although most studies about mediation refer to general exercise treatments they are very valuable in demonstrating the need for physical activity in everyday life regarding the preservation of function and prevention of pain.

### Who Comes First: Moderation or Mediation

Regarding the literature it is obviously that psychosocial factors significantly influence the effectiveness of pain management interventions. But till now, it is still unclear whether this is based on moderation or mediation. The presented results suggest that the expression of pain intensity due to different interventions is moderated by psychosocial factors, which was elaborated here very specifically for sensorimotor training and strict controlled (baseline pain, age, gender physical activity in general) and which can be well-justified by the addressed underlying physiological processes of the treatment forms. Moreover, a stronger moderation can be seen predominantly short-term and lasts mainly over the active period-i.e. during continuous training execution. This also fits well with existing literature, which suggests that pain intensity alter more rapidly by interventions than disability or function. The moderation of exercise effects on pain intensity and disability decrease over time in presented data, although we further still do not see a mediation. Mediation processes may play role in their long-term influence on physical activity and psychosomatic symptoms such as depression in general, but they are strongly conflicted by lifestyle factors and methodologically difficult to extract, which was not study objective here.

### Limitations

First, for mediation no non-linear regression techniques to model the intervention-mediator and mediator-outcome effects were explored. Further, no use of latent growth modeling which allows inclusion of multiple time points (at least 3 measurements over different time points), providing a more accurate estimate of change both within and between participants (75). However, all mediation analysis were controlled for baseline measures of pain and respective mediators. Second, sample size calculation was considered for testing treatment main effects (e.g., intention to treat analysis), which was not study objective here. As the analysis of pathways would be strongly afflicted by imputation of missing cases (e.g., missing measurement point) we preferred a complete case analysis and controlled further for age, gender, baseline pain, and physical activity in general which might be a strength of this study. However, this reduction of the sample size may limit the statistical power (e.g., exclusion of athletes, inclusion of four study-centers). Third, participant could not be blinded, although study personal was (single blinding). Fourth, due to the high number of statistical tests in moderation and mediation analyses multiple testing problems substantially increase the probability of incorrectly rejecting null hypotheses. Finally, effect sizes of the exercise intervention on pain were low (max. *d* = 0.2, although this is typical for exercise intervention studies) (72).

## Conclusion

The results regarding a possible moderation of the response to a sensorimotor-exercise-intervention on LBP are of high relevance and raise hope for an explanation of the long-lasting heterogeneity of exercise effects on CLBP. They provide insight into a possible interaction between pain and exercise, and its dose-response relationship. Further research in this area is needed for the development of diagnostic tools (76, 77) and personalized therapy modules with reinforcing effects for self-management care (35). This would lead to a great benefit for future therapy and prevention concepts.

## Data Availability Statement

The datasets for this study can be found in the MiSpEx Network, the doi is requested at University of Potsdam.

## Ethics Statement

All clinical investigations have been conducted according to the principles expressed in the Declaration of Helsinki. Final ethical approval has been provided 01/25/2012 by the major institutional ethics review board of the University of Potsdam, Germany (number 36/2011). The study was registered as a clinical trial 05/16/2013 in the German Clinical Trial Register with the identification number: DRKS00004977. Study conduction between 06/2013-12/2014. All adverse events were reported in the manuscript.

## Author Contributions

P-MW: writing original draft. DN: editing. P-MW and FM: conceptualization and data curation. HB, MS, CS, WB, and FM: investigation. DD and P-MW: methodology and formal analysis. All authors: resources. FM: funding, prinicipal investigator (PI), and project administration.

## Conflict of Interest

The authors declare that the research was conducted in the absence of any commercial or financial relationships that could be construed as a potential conflict of interest.

## Publisher's Note

All claims expressed in this article are solely those of the authors and do not necessarily represent those of their affiliated organizations, or those of the publisher, the editors and the reviewers. Any product that may be evaluated in this article, or claim that may be made by its manufacturer, is not guaranteed or endorsed by the publisher.

## Figures and Tables

**Table:** 

CPG	Chronic Pain Grade Questionnaire
CPI	Characteristic Pain intensity, scale of Chronic Pain Grade questionnaire
BSSS	Berliner Social Support Questionnaire
DISS	Subjective pain disability, scale of Chronic Pain Grade questionnaire
DRKS	German Clinical Trials Register
GH	General Health questions (standardized)
FABQ-D	Fear-Avoidance Questionnaire
HADS	Hamilton Anxiety and Depression Scale
LBP/CLBP	Low Back Pain/ Chronic Low Back Pain
M1, M2, M3, M4, M5	Visits 1-5
MiSpEx	The German Research Network of Medicine in Spine Exercise
MBSR	Mindfulness Based Stress Reduction Program
N	Number of participants
POMS	Profile of Mood Questionnaire
p -values	Significance level: *p* < 0.01, *p* < 0.05, or *p* < 0.10
PSS	Perceived Stress Scale
PVAQ	Pain Vigilance Avoidance Questionnaire
Sleep	Standardized questions about sleeping routines
SMT	Sensorimotor training
SMT-BT	Sensorimotor training with behavioral therapy elements (Body Scan of MBSR, working memory task during exercising, e.g., n-back, Psychoeducation)
VE	Vital Exhaustion

## Appendix B. Intervention

The training was supervised for a 3-week center-based period by therapists, followed by a 9-week self-administered home-based training by DVD. Participants were scheduled to train three times a week (30 min duration, four different sensorimotor exercises: Basic: (1) quadrupedal/all-fours stability, (2) deadlift/rowing, (3) double leg – single leg heel-pad-stance and (4) side planks, three series of 10 repetitions, 2 min break). For individualization purposes, all exercises comprised 12 different levels of difficulty (at higher level with additional weights). Beyond this standard program, the SMT group received a standardized stretching program and SMT-BT group three additional behavioral modules (“cognitive distraction during exercising,” “Body Scan-Unit,” and “Psychoeducation” after exercising). Home-based training adherence was monitored by a self-administered training log, kept by the participant. For details, please see (35, 78) and further (79).
